# Reducing Aggression and Improving Physical Fitness in Adolescents Through an After-School Volleyball Program

**DOI:** 10.3389/fpsyg.2020.02081

**Published:** 2020-08-12

**Authors:** Nebojša Trajković, Maja Pajek, Goran Sporiš, Lidija Petrinović, Špela Bogataj

**Affiliations:** ^1^Faculty of Sport and Physical Education, University of Novi Sad, Novi Sad, Serbia; ^2^Faculty of Sport, University of Ljubljana, Ljubljana, Slovenia; ^3^Faculty of Kinesiology, University of Zagreb, Zagreb, Croatia; ^4^Department of Nephrology, University Medical Centre, Ljubljana, Slovenia

**Keywords:** sport, aggression, adolescents, fitness, physiological characteristics, high school

## Abstract

This study aimed to determine the effects of an after-school volleyball program on aggression and physical fitness in 14–16 years old students. One hundred and seven participants were randomized to a small-sided volleyball (SSV) training group or a control group (CON). The SSV group completed 8 months of small-sided volleyball training twice a week after school in addition to the regular physical education classes. Yo-Yo Intermittent Recovery Level 1 test (YYIRT1), medicine ball throw (MED), vertical jump (VJ), and Buss and Perry’s aggression questionnaire were evaluated before and after 8 months of training. Results revealed a significant interaction effect (group × time) in total sample for physical aggression [*F*(1, 105) = 17.688; *p* < 0.001], verbal aggression [*F*(1, 105) = 4.973; *p* = 0.028], anger [*F*(1, 105) = 7.662; *p* = 0.007], medicine ball throw [*F*(1, 105) = 36.143; *p* < 0.001], and YYIRT1 [*F*(1, 105) = 12.508; *p* = 0.001]. After-school small-sided volleyball for adolescents was accompanied by a significant decrease in aggression compared to physical education classes only. Additionally, adolescents from SSV group showed better results in physical fitness compared to the control group. Our findings significantly contribute to the understanding of possible mechanisms for reducing adolescents’ aggression, which include enjoyment, motivation, and self-control through sport intervention.

## Introduction

Physically active adults have less chronic diseases and lower rates of premature death compared to their inactive counterparts (Bauman, 2004; Physical Activity Guidelines for Americans, 2008). The development of the mentioned conditions is already manifested in childhood and adolescence (Cook et al., 2009; Halfon et al., 2012; Kohl and Cook, 2013). Therefore, the promotion of a healthy and active lifestyle should start in our early life. It is also known that childhood and adolescent physical activity influence on adult morbidity and mortality (Hallal et al., 2006). People create their adulthood lifestyle patterns as they go through adolescence (Hallal et al., 2006). Besides physical inactivity, aggressive behavior is one of the main public health concerns among adolescents (Zinatmotlagh et al., 2013). There are many theories that could explain the occurrence of aggressive behavior in children and adolescents. According to Bjorkly (2006), aggressive behavior should be examined through three main groups of aggression theories: psychoanalytic, drive, and learning theory. The most common is the social learning theory (Bandura, 1978) that explains aggressive behavior in children and adolescents by observing other’s behavior. One of the most used instruments for measuring aggressive behavior in adolescents has been constructed by Buss and Perry (1992). This model of aggression represents the tendency to respond aggressively when facing rejection, stress, or difficulties (Shachar et al., 2016). The authors grouped the aggression into four factors: physical aggression, verbal aggression, anger, and hostility, combining cognitive, emotional, and behavioral components. Although each factor explained unique variance, this four-factor structure of the questionnaire sheds light on the specific manifestations of trait aggression (Eastin, 2013).

Innumerable attempts have been made in order to reduce aggression in adolescents (Castillo-Eito et al., 2020). School-based physical activity interventions are practical, effective, and universally applicable (Kriemler et al., 2011; Rodríguez et al., 2016). They were proven to have positive effects on physical fitness and health markers (Dobbins et al., 2013; Sun et al., 2013; Lai et al., 2014). Doubling the frequency of physical education sessions resulted in physical fitness improvement, especially aerobic fitness, which is linked to adolescents’ cardiovascular health (Ardoy et al., 2011). One semester of aerobic exercise classes showed significant improvement in cardiorespiratory fitness and a decrease in systolic blood pressure in ninth-grade girls (Ewart et al., 1998). Furthermore, research regarding the psychological benefits of exercise-based school interventions is limited.

Team games (sports) have recently been used to determine the impact on different physical fitness components in the school population (Seabra et al., 2014; Cvetković et al., 2018; Trajković et al., 2020a). Small-sided recreational football has been recognized as one of the most popular and effective team sport in adolescents (Hammami et al., 2018). According to the authors’ knowledge, up to date, no study exists that investigated the effects of small-sided games in volleyball on physical fitness in children and adolescents. Moreover, there are only a few studies concerning the impact of sport participation on aggressive behavior in adolescents. Aggression can occur in many forms, from verbal and social aggression to physical assault and violence (Karriker-Jaffe et al., 2008). Typically, aggressive behaviors increase their severity in adolescent development (Loeber and Hay, 1997; Tolan et al., 2000). Therefore, interventions that could decrease aggression in adolescents are highly needed. After-school physical activity (e.g., basketball, football, volleyball, martial arts, capoeira) carried out five times a week, significantly reduced anger, hostile thoughts, physical aggression, and negative emotions in 8–12 years old children (Shachar et al., 2016). The implementation of small-sided recreational football into traditional physical education classes showed to be a beneficial approach for lowering aggression and improving the physical fitness of high-school students (Trajković et al., 2020a). However, some sports of aggressive nature showed the opposite results (Mason and Wilson, 1988; Monacis et al., 2015). This was confirmed by Kreager (2007), who stated that playing contact sports (e.g., football and wrestling) could subsequently lead to violence. Therefore, it would be interesting to see if sports that have no direct physical contact could transmit their learned behavior on the field into off-sport situations.

Additionally, one study showed that participation in sporting activity was associated with an increase in delinquent behavior in adolescents (Begg, 1996). Involvement in competitive sports games increased children’s levels of aggression, despite the final result (Nucci and Young-Shim, 2005). Regardless of inconsistent findings, physical activity is still recommended as a method for reducing aggression. One approach that could connect sport participation and aggression is through self-control skills (SCS) (Shachar et al., 2016). The link between sports and SCSs was explained earlier (Marsh and Kleitman, 2003; Miller et al., 2005). The authors mention several mechanisms in sport that could contribute to better SCS. Sports games require attention, focus, and concentration because of the movements and actions that must be performed in a specific order with precise timing as well as because of the compliance with laws and regulations (Marsh and Kleitman, 2003; Miller et al., 2005). Moreover, actions in team sports include mostly the problem-solving situations because while playing, children must make rapid decisions under pressure and think consciously, creatively, and quickly (Marsh and Kleitman, 2003; Miller et al., 2005). Additionally, it was demonstrated that sports activities are positively linked with positive emotions (Chantal et al., 2005; Hyde et al., 2011; Monacis et al., 2014).

In the current study, we expected that changes in enjoyment and positive emotions following school sports intervention would lead to a decrease in the participants’ aggressive behavior. The present study aims to determine the effect of after school small-sided volleyball on aggression and physical fitness in 14–16 years old students. It was hypothesized that involvement in small-sided volleyball game would reduce aggression and increase physical fitness.

## Materials and Methods

### Subjects

One hundred and seven adolescents from different classes in school were included in the study, of whom 56 [*n* = 17 girls and 38 boys; age = 15.5 ± 0.7 years; years to and from peak height velocity (Y-PHV) = 0.4 ± 0.7 years] were randomized to a small-sided volleyball training group (SSV) and 51 (*n* = 19 girls and 35 boys; age = 15.7 ± 0.6 years; Y-PHV = 0.5 ± 0.9 years) to a control group (CON) that maintained their usual physical-education activities. There were no time-loss injuries during the volleyball program. The maturity of participants was estimated by predicting age at peak height velocity (PHV) (Malina et al., 2015). All participants were informed verbally and in writing about the procedures and possible risks and agreed to participate in the study with the signed informed consent of their parents or guardians. The study was conducted according to the Declaration of Helsinki, and the ethical approval for the study was obtained from the ethics committee of the Faculty of Sport and Physical Education at the University of Novi Sad (Ref. no. 12/2018).

### Procedures

The study was performed in the period from September 2018 to May 2019. Following a series of familiarization sessions, the first week was used for pre-intervention assessment for both the small-sided volleyball group and the control group. In months 1–8, the SSV group completed 64 volleyball sessions after school: two scheduled ∼45-min physical-education sessions per week separated by at least 1 day. SSV group had also attended regular physical-education classes. In the same period, the CON group undertook their regular physical-education classes (handball, basketball, gymnastics, table tennis, athletics). Finally, by the end of the 8 months, students from both the SSV and control group completed the same tests as before week 1. In Serbia, it is compulsory for school children to have two 45 min classes of PE per week. Trained university staff members have performed all the necessary tests with a requirement that no physical activity could be performed on the day before the test day 1.

The test day 1 included body mass and weight, vertical jump, and medicine ball throw, while test day 2 included the Aggression Questionnaire and Yo-Yo intermittent recovery level 1 test (YYIRT1). Before testing, the participants performed a standardized 10-min warm-up consisting of jogging, dynamic stretching, and running drills.

### Testing

Medicine ball throw (Gabbett et al., 2006). The participant held a 2-kg rubber medicine ball (Tigar, Pirot, Serbia) with arms behind head, and throwing the ball over the head straight forward as far as possible. The result is the distance from the front of the line to the point where the ball landed recorded to the nearest 1 cm. Three attempts were made. The best of the three attempts, with a 1 min rest interval between each attempt, was used for further analysis. The intraclass correlation coefficient (ICC) and the coefficient of variation (CV) for measuring medicine ball throw were 0.832 and 3.84%, respectively.

Vertical jump (countermovement jump). We used the Optojump (Optojump photocell system; Microgate, Italy) to measure vertical jump performance. The countermovement jump (CMJ) with arms was chosen as the simplest jump and the most similarities with volleyball jumps. The subjects were instructed to start from the upright position and to perform a CMJ with arm swing during the execution of the jump (i.e., hands were free to move). It was highly recommended for the participants that during their take-off, they leave the floor with the ankles and knees extended and land in a, similarly, extended position. The rest interval between jump repetitions was 30 s. The ICC and CV for vertical jump performance were 0.911 and 2.89%.

Yo-Yo Intermittent Recovery Level 1 test (YYIRT1) was performed according to the guidelines established by Póvoas et al. (2016). YYIRT1 performance and associate measured heart rate (HR) peak are reliable for 9–16 years-old soccer players and non-sports-active boys (Póvoas et al., 2016). Briefly, the participants during YYIRT1 must repeatedly run 2 × 20-m back and forth (180-degree turns) between the starting, turning, and the finishing line at a progressively increased speed. The speed was controlled by beeps from an audio device. The test was completed when the participants fail to complete a shuttle within the given signal twice, and the final score is the last shuttle the player has completed. For further analysis, we have used the total distance (meters).

The Aggression Questionnaire was used to assess aggressive behavior (Buss and Perry, 1992). It consists of 29 items that determine four dispositional subtracts of aggression, physical aggression, verbal aggression, anger, and hostility. A five-point Likert scale was used: 5 points (from Extremely uncharacteristic of me = 1 to Extremely characteristic of me = 5) that can be further aggregated for a general aggression score or calculated separately: The reliability of the instrument in the adolescent population was previously verified (Santisteban and Ma Alvarado, 2009). The internal consistency coefficient (Cronbach’s alpha) was 0.88 for physical aggression, 0.89 for verbal Aggression, 0.82 for anger, and 0.84 for hostility.

### Training Program

The small-sided volleyball was played on smaller volleyball courts. A court size (4.5–6 m in width, 9–12 m in length) was used for playing volleyball 3 vs. 3 and 4 vs. 4 players. Each session consisted of a warm-up lasting ∼10 min [moderate-intensity running (2 min), dynamic stretching (4 min), and specific volleyball warm up with the ball (4 min)], ∼30 min of volleyball and ∼5 min of cool-down. The intensity of training was monitored using the Polar Team System H7 (Polar Electro Oy, Kempele, Finland). The same eight players were monitored for the first 2 weeks and the last 2 weeks of the training program. The perceived exertion was measured using the rate of perceived exertion (RPE) scores (10-point scale) collected 30 min after the end of the sessions during the training period (Foster et al., 2001). Furthermore, we compared the physiological responses and enjoyment between training sessions involving the small-sided volleyball or physical education (PE) classes. A modified Physical Activity Enjoyment Scale (PACES) was completed after the SSV and PE classes (Motl et al., 2001). The PACES consists of a 16-item questionnaire relating, and it uses a 5-point Likert-scale.

The control group undertook their regular PE classes involving ball games (soccer, handball, basketball), instructional training, and individual sports common to many European countries (gymnastics, table tennis, athletics), excluding volleyball. Each type of sports activity lasted around 1 month. In the control group, HR was also monitored during PE classes. Both groups were not engaged in other organized physical activities besides small-sided volleyball and PE classes.

### Statistical Analysis

Data analysis was carried out using SPSS, version 22 (SPSS Inc., Chicago, IL, United States). Mean ± standard deviation was determined for all outcome measures. The normality of data was checked using the Kolmogorov–Smirnov test and demonstrated that all data had a normal distribution (*p* > 0.05). Moreover, Levene’s tests were determined for all psychological outcomes and physical test variables. Test-retest reliability for variables was assessed using the ICC and CV. A two way repeated measures ANOVA (2 × 2) was computed to test for interactions and main effects for time (pre-test vs. post-test) and group (SSV vs. CON) on the selected psychological outcomes and physical test variables., The effect size (ES) of each variable was tested using Cohen’s d within each group and was classified as follows: <0.2 was defined as trivial; 0.2–0.6 was defined as small; 0.6–1.2 was defined as moderate; 1.2–2.0 was defined as large; >2.0 was defined as very large, and >4.0 was defined as extremely large (Hopkins et al., 2009). Additionally, a partial eta (η) squared was used for difference between groups (0.01 = small effect, 0.06 = medium effect, and 0.14 = large effect) (Pallant, 2007). Statistical significance was set at *p* ≤ 0.05.

## Results

**FIGURE 1:**
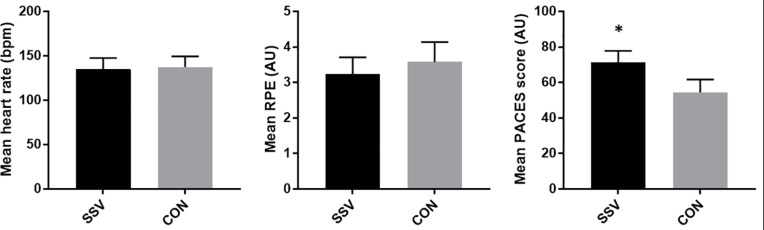
Mean heart rate, mean RPE, and mean PACES score in SSV and CON group during the intervention. RPE, rate of perceived exertion; PACES, Physical Activity Enjoyment Scale; SSV, small-sided volleyball group; CON, control group; *significantly different from the CON group.

For the SSV group, mean HR and RPE during training were 134.6 ± 12 bpm and 3.2 ± 0.5 AU (arbitrary unit), respectively. The CON group showed similar results for mean HR and RPE (137.4 ± 13bpm; 3.58 ± 0.6 AU). Significant differences (*p* < 0.001) were observed for mean PACES between SSV and CON group (Figure 1).

Table 1 presents results from both groups (boys and girls together). Results revealed a significant effect of group (SSV vs. CON) x time (pre to post) interaction for pagression [*F*(1, 105) = 17.688; *p* < 0.001], vagression [*F*(1, 105) = 4.973; *p* = 0.028], anger [*F*(1, 105) = 7.662; *p* = 0.007], medicine ball throw [*F*(1, 105) = 36.143; *p* < 0.001], and YYIRT1 [*F*(1, 105) = 12.508; *p* = 0.001]. Along with the mentioned significance, there was a significant main effect for time in hostility [*F*(1, 105) = 22.359; *p* < 0.001], and CMJ [*F*(1, 105) = 17.652; *p* < 0.001].

**TABLE 1:** Changes in physical fitness and aggression measures from pre- to post-test in SSV and CON group for the total sample.

**Group**	**Pre-test**	**Post-test**	**ES**	**% change**	***p*-value, η^2^*_*p*_***
**Pagression**					
SSV	29.93 ± 5.20	26.63 ± 4.89	−0.65	−11.0%	Group: *p* = 0.053, η^2^*_*p*_*: 0.036 Time: *p* < 0.001, η^2^*_*p*_*: 0.296 Interaction: *p* < 0.001, η^2^*_*p*_*: 0.147
CON	26.55 ± 6.55	25.82 ± 6.01	−0.12	−2.7%	
**Vagression**					
SSV	18.50 ± 2.98	17.26 ± 3.66	−0.37	−6.7 %	Group: *p* = 0.306, η^2^*_*p*_*: 0.010 Time: *p* = 0.004, η^2^*_*p*_*: 0.079 Interaction: *p* = 0.028, η^2^*_*p*_*: 0.046
CON	18.69 ± 4.22	18.51 ± 4.18	−0.04	−1.0%	
**Hostility**					
SSV	25.96 ± 5.13	23.87 ± 5.72	−0.38	−8.1%	Group: *p* = 0.562, η^2^*_*p*_*: 0.003 Time: *p* < 0.001, η^2^*_*p*_*: 0.178 Interaction: *p* = 0.083, η^2^*_*p*_*: 0.029
CON	26.02 ± 5.99	25.06 ± 6.03	−0.16	−3.7%	
**Anger**					
SSV	18.91 ± 4.25	17.20 ± 4.43	−0.39	−9.0%	Group: *p* = 0.036, η^2^*_*p*_*: 0.042 Time: *p* < 0.001, η^2^*_*p*_*: 0.249 Interaction: *p* = 0.007, η^2^*_*p*_*: 0.069
CON	20.08 ± 4.20	19.47 ± 4.21	−0.15	−3.0%	
**CMJ**					
SSV	36.61 ± 6.27	37.69 ± 6.24	+0.17	+3.0%	Group: *p* = 0.293, η^2^*_*p*_*: 0.011 Time: *p* < 0.001, η^2^*_*p*_*: 0.146 Interaction: *p* = 0.172, η^2^*_*p*_*: 0.018
CON	35.40 ± 8.12	35.95 ± 8.17	+0.07	+1.6%	
**Medicine ball throw**					
SSV	8.63 ± 1.74	9.08 ± 1.75	+0.26	+5.2%	Group: *p* = 0.034, η^2^*_*p*_*: 0.043 Time: *p* < 0.001, η^2^*_*p*_*: 0.160 Interaction: *p* < 0.001, η^2^*_*p*_*: 0.260
CON	8.17 ± 1.81	8.10 ± 1.64	−0.05	−0.9%	
**YYIRT1**					
SSV	1464.07 ± 120.21	1499.63 ± 121.53	+0.29	+2.4%	Group: *p* = 0.026, η^2^*_*p*_*: 0.047 Time: *p* < 0.001, η^2^*_*p*_*: 0.274 Interaction: *p* = 0.001, η^2^*_*p*_*: 0.108
CON	1422.35 ± 134.62	1432.16 ± 125.93	+0.08	+0.7%	

**Paggression, physical aggression; Vagression, verbal aggression; SSV, small-sided volleyball group; CON, control group; ES, Cohen d effect size; CMJ, countermovement jump; YYIRT1, Yo-Yo intermittent recovery test level 1.**

Results for boys only are presented in Table 2. Pagression [*F*(1, 71) = 12.065; *p* = 0.001], medicine ball throw [*F*(1, 71) = 40.311; *p* < 0.001], and YYIRT1 [*F*(1, 71) = 12.727; *p* = 0.001] demonstrated the significant group (SSV vs. CON) × time (pre to post) interaction. The significant main effect for time was found for vagression [*F*(1, 71) = 4.650; *p* = 0.035], hostility [*F*(1, 71) = 11.964; *p* = 0.001], anger [*F*(1, 71) = 22.690; *p* < 0.001], and CMJ [*F*(1, 71) = 11.871; *p* = 0.001].

**TABLE 2:** Changes in physical fitness and aggression measures from pre- to post-test in SSV and CON group for boys.

**Group**	**Pre-test**	**Post-test**	**ES**	**% change**	***p*-value, η^2^*_*p*_***
**Pagression**					
SSV	30.37 ± 5.45	27.16 ± 5.13	−0.61	−10.6%	Group: *p* = 0.011, η^2^*_*p*_*: 0.09 Time: *p* < 0.001, η^2^*_*p*_*: 0.289 Interaction: *p* = 0.001, η^2^*_*p*_*: 0.149
CON	25.48 ± 7.08	24.82 ± 6.38	−0.09	−2.6%	
**Vagression**					
SSV	18.61 ± 2.91	17.61 ± 3.88	−0.29	−5.4%	Group: *p* = 0.895, η^2^*_*p*_*: 0.000 Time: *p* = 0.035, η^2^*_*p*_*: 0.063 Interaction: *p* = 0.222, η^2^*_*p*_*: 0.022
CON	18.12 ± 4.57	17.85 ± 4.58	−0.06	−1.5%	
**Hostility**					
SSV	25.89 ± 4.99	24.03 ± 5.93	−0.34	−7.2%	Group: *p* = 0.750, η^2^*_*p*_*: 0.001 Time: *p* = 0.001, η^2^*_*p*_*: 0.148 Interaction: *p* = 0.148, η^2^*_*p*_*: 0.030
CON	25.79 ± 6.74	25.03 ± 6.79	−0.11	−2.9%	
**Anger**					
SSV	19.50 ± 4.30	18.00 ± 4.26	−0.35	−7.7%	Group: *p* = 0.228, η^2^*_*p*_*: 0.021 Time: *p* < 0.001, η^2^*_*p*_*: 0.247 Interaction: *p* = 0.058, η^2^*_*p*_*: 0.051
CON	20.30 ± 4.48	19.67 ± 4.47	−0.14	−3.1%	
**CMJ**					
SSV	39.15 ± 4.37	40.22 ± 4.56	+0.24	+2.7%	Group: *p* = 0.328, η^2^*_*p*_*: 0.014 Time: *p* = 0.001, η^2^*_*p*_*: 0.147 Interaction: *p* = 0.329, η^2^*_*p*_*: 0.014
CON	40.42 ± 4.65	41.01 ± 4.41	+0.13	+1.5%	
**Medicine ball throw**					
SSV	9.46 ± 1.13	9.92 ± 1.04	+0.42	+4.9%	Group: *p* = 0.047, η^2^*_*p*_*: 0.056 Time: *p* = 0.029, η^2^*_*p*_*: 0.067 Interaction: *p* < 0.001, η^2^*_*p*_*: 0.369
CON	9.31 ± 0.99	9.08 ± 1.03	−0.23	−2.5%	
**YYIRT1**					
SSV	1513.68 ± 97.77	1551.58 ± 99.36	+0.38	+2.5%	Group: *p* = 0.119, η^2^*_*p*_*: 0.035 Time: *p* < 0.001, η^2^*_*p*_*: 0.224 Interaction: *p* = 0.001, η^2^*_*p*_*: 0.156
CON	1493.33 ± 105.20	1497.58 ± 101.71	+0.04	+0.3%	

**Paggression, physical aggression; Vagression, verbal aggression; SSV, small-sided volleyball group; CON, control group; ES, Cohen d effect size; CMJ, countermovement jump; YYIRT1, Yo-Yo intermittent recovery test level 1.**

Results for girls only are presented in Table 3.

**TABLE 3:** Changes in physical fitness and aggression measures from pre- to post-test in SSV and CON group for girls.

**Group**	**Pre-test**	**Post-test**	**ES**	**% change**	***p*-value, η^2^*_*p*_***
**Pagression**					
SSV	28.88 ± 4.53	25.38 ± 4.13	−1.09	−12.1%	Group: *p* = 0.529, η^2^*_*p*_*: 0.012 Time: *p* = 0.001, η^2^*_*p*_*: 0.310 Interaction: *p* = 0.026, η^2^*_*p*_*: 0.145
CON	28.50 ± 5.06	27.67 ± 4.91	−0.17	−2.9%	
**Vagression**					
SSV	18.25 ± 3.21	16.44 ± 3.03	−0.80	−9.9%	Group: *p* = 0.026, η^2^*_*p*_*: 0.146 Time: *p* = 0.034, η^2^*_*p*_*: 0.132 Interaction: *p* = 0.034, η^2^*_*p*_*: 0.132
CON	19.72 ± 3.37	19.72 ± 3.10	0.00	0.0%	
**Hostility**					
SSV	26.13 ± 5.62	23.50 ± 5.38	−0.66	−10.1%	Group: *p* = 0.549, η^2^*_*p*_*: 0.011 Time: *p* = 0.003, η^2^*_*p*_*: 0.243 Interaction: *p* = 0.304, η^2^*_*p*_*: 0.033
CON	26.44 ± 4.42	25.11 ± 4.50	−0.30	−5.0%	
**Anger**					
SSV	17.50 ± 3.90	15.31 ± 4.39	−0.53	−12.5%	Group: *p* = 0.028, η^2^*_*p*_*: 0.142 Time: *p* = 0.002, η^2^*_*p*_*: 0.269 Interaction: *p* = 0.049, η^2^*_*p*_*: 0.115
CON	19.67 ± 3.73	19.11 ± 3.77	−0.15	−2.8%	
**CMJ**					
SSV	30.58 ± 6.07	31.69 ± 5.63	+0.19	+3.6%	Group: *p* = 0.009, η^2^*_*p*_*: 0.196 Time: *p* = 0.025, η^2^*_*p*_*: 0.147 Interaction: *p* = 0.342, η^2^*_*p*_*: 0.028
CON	26.20 ± 3.83	26.67 ± 4.33	+0.11	+1.8%	
**Medicine ball throw**					
SSV	6.67 ± 1.30	7.08 ± 1.43	+0.30	+6.1%	Group: *p* = 0.075, η^2^*_*p*_*: 0.096 Time: *p* < 0.001, η^2^*_*p*_*: 0.492 Interaction: *p* = 0.088, η^2^*_*p*_*: 0.088
CON	6.08 ± 0.80	6.30 ± 0.76	+0.28	+3.6%	
**YYIRT1**					
SSV	1346.25 ± 80.24	1376.25 ± 68.98	+0.40	+2.2%	Group: *p* = 0.017, η^2^*_*p*_*: 0.167 Time: *p* < 0.001, η^2^*_*p*_*: 0.404 Interaction: *p* = 0.358, η^2^*_*p*_*: 0.026
CON	1292.22 ± 68.99	1312.22 ± 60.25	+0.31	+1.5%	

**Paggression, physical aggression; Vagression, verbal aggression; SSV, small-sided volleyball group; CON, control group; ES, Cohen d effect size; CMJ, countermovement jump; YYIRT1, Yo-Yo intermittent recovery test level 1.**

Results for girls indicated a significant group (SSV vs. CON) × time (pre to post) interaction effect for pagression [*F*(1, 34) = 5.437; *p* = 0.026], vagression [*F*(1, 34) = 4.881; *p* = 0.034], and anger [*F*(1, 34) = 4.176; *p* = 0.049]. A significant main effect for time was found for hostility [*F*(1, 34) = 10.265; *p* = 0.003], CMJ [*F*(1, 34) = 5.500; *p* = 0.025], medicine ball throw [*F*(1, 34) = 31.000; *p* < 0.001], and YYIRT1 [*F*(1, 34) = 21.719; *p* < 0.001].

## Discussion

Focusing on aggressive behavior in high school children, the current study attempted to demonstrate the effectiveness of after school sports activity intervention for reducing aggressive behavior and improving physical fitness. The major findings were that adolescents from the experimental group after 8 months of after-school volleyball activities, two times per week, reported larger reductions in aggression, in comparison to adolescents from the control group. Additionally, adolescents from small-sided volleyball group showed better results in physical fitness compared to the control group. Moreover, our findings demonstrate that girls showed larger reductions in aggression compared to boys with similar improvements in physical fitness.

Previous after school exercise interventions proved to be a significant factor in reducing aggressive behavior in adolescents (Eccles et al., 2003; Park et al., 2017). There are only a few studies targeting youth psychological outcomes and team sport exercise interventions (Seabra et al., 2014; Shachar et al., 2016; Hammami et al., 2018; Trajković et al., 2020a). However, most of the mentioned studies included recreational football with different formats, with only one intervention that included different sports activities (Shachar et al., 2016). Shachar et al. (2016) found that after-school sports activities (e.g., basketball, soccer, volleyball, martial arts, capoeira) produce larger reductions in anger, hostile thoughts, physical aggression, and negative emotions in comparison to a regular PE classes in children 8–12 years of age. Concerning adolescents, Trajković et al. (2020a) showed a significant decrease in physical aggression among high-school students participating in the 8-month recreational soccer program compared to PE classes, which is similar to our results if we look at the total sample. Besides physical aggression, our results showed a significant reduction in verbal aggression and anger. Our results support the fact that those who are engaged in team sports have the potential to promote teamwork, sharing, and better interpersonal relationships with peers and adults, which may significantly contribute to enhancing psychological status (Pedersen and Seidman, 2004). Additionally, compared to other team sports, there is no direct physical contact in volleyball that could lead to the use of physical violence more often (Mutz, 2012). The current study showed that adolescents could transmit their learned behavior on the field into off-sport situations.

Different models were proposed in order to reduce the level of aggression or to improve the management of aggressive behavior. One proven model that was showed to be beneficial in reducing the level of aggression is through indirect mechanisms with the gains in self-control skills in adolescents (Ronen et al., 2007). The research showed that self-control skills were mediating mechanisms that led to reductions in aggressive behavior (Shachar et al., 2016). Moreover, Park et al. (2017) stated that after school sports activities show many psychological advantages by encouraging self-effacement, mutual respect, and consideration. The participants in the current study enrolled in small-sided volleyball showed greater enjoyment compared to participants enrolled in PE classes only (Figure 1), which could be the reason for a greater reduction in aggressive behavior. A recent study stated that the differences between male and female adolescents should be taken into account (Park et al., 2017). Unfortunately, a novel study that included team sport intervention did not show the analysis by gender (Trajković et al., 2020a). Nevertheless, Shachar et al. (2016) showed that physical aggression in girls is changed directly through changes in self-confidence skills. In contrast, changes in self-confidence skills in boys were linked indirectly to changes in physical aggression, through changes in positive and negative emotions. This was confirmed in our study, where boys and girls showed different results for reduction in aggressive behavior. Boys showed an only significant reduction in physical aggression compared to PE classes, while girls significantly reduced physical aggression, verbal aggression, and anger. It was demonstrated that adolescents’ aggression decreased over time (Park et al., 2017). Therefore, a possible discrepancy in results between male and female adolescents could be due to the type of intervention, initial status, and slope.

Due to the fact that PE does not provide sufficient opportunities for children and adolescents to achieve the provided recommendations, there has been an increase in the number of after-school physical activity programs (Demetriou et al., 2017). It was demonstrated in previous studies that after-school exercise programs with fitness emphasis are effective for improving physical fitness in children and adolescents (Ara et al., 2006; Gutin et al., 2008; Carrel et al., 2011). Recreational team sports provide fun and effective options to encourage adolescents to improve their fitness profile (Larsen et al., 2017; Cvetković et al., 2018; Hammami et al., 2018; Trajković et al., 2020a). However, there was only limited positive fitness effects following frequent low-volume ball games and interval running in children aged 8–10 years (Larsen et al., 2017). On the contrary, greater improvements were found in physical fitness following recreational football intervention in children and adolescents (Cvetković et al., 2018; Hammami et al., 2018; Trajković et al., 2020a).

In the current study, vertical jump (CMJ) was improved by 3.0% (ES = 0.17) after 8 months of recreational small-sided volleyball training; however, without significant differences between groups. The improvement in vertical jump was expected, having in mind that players in volleyball perform more than 250 jumps in a volleyball match (Martinez, 2017). However, there was a smaller improvement in vertical jump performance in recreational settings, probably due to lower intensity in recreational volleyball (72% HR max and 3.14 RPE) (Trajković et al., 2020b). This was confirmed in a recent study in recreational football training (78–84% HR max) with significantly greater effects on countermovement jumping (17.0%; ES = 0.76, moderate) in overweight and obese children (Cvetković et al., 2018). On the contrary, Hammami et al. (2018) found only limited effects on jump performance in untrained adolescents, following 8 weeks of soccer training. Two studies have used volleyball programs in school students (Sozen, 2012; Selmanović et al., 2013). However, in both studies standing long jump was evaluated after interventions, with contrary results.

The present study found improvement in medicine ball throw in the total sample. Boys showed similar improvements compared to girls following 8 months of small-sided volleyball (4.9%; ES = 0.42 vs. 6.1%; ES = 0.30). In a similar study, Gabbett et al. (2006) found improvements in agility and speed but not in medicine ball throw in adolescent boys following 8 weeks of skill-based training. However, the authors stated that similar pre-training and post-training results for medicine ball throw might be attributed to the low intensity during skill-based training. On the contrary, Gabbett (2008) found greater improvements for medicine ball throw in adolescent boys and girls after skill-based conditioning games, which was due to the higher percentage of time spent at heart rates greater than 85%. The present study had a lower intensity compared to the study of Gabbett (2008), but with a much longer duration of the study (8 months compared to 12 weeks).

There is well-documented evidence that an increase in cardiorespiratory fitness could reduce the risk of developing cardiovascular diseases and premature mortality (Lee and Skerrett, 2001). A major finding in the current study was that cardiorespiratory fitness significantly increased (2.4%) with small-sided volleyball, with no significant changes observed in the control group. Our results are similar to those documented in a recent review, which stated that group sports intervention improves cardiorespiratory fitness (pooled ES = 0.53) in overweight and obese children (Oliveira et al., 2017). Our results are also in line with previous studies performed in children and adolescents following recreational soccer programs (Vasconcellos et al., 2016; Hammami et al., 2018; Trajković et al., 2020a). Most of the published studies that involved group sports mainly focus on aerobic activities (e.g., football, basketball, handball) with moderate to high intensity, which is beneficial in improving VO_2_peak (Oliveira et al., 2017). Two studies with similar programs were conducted in adolescents already enrolled in volleyball training (Gabbett et al., 2006; Gabbett, 2008). The first study (Gabbett et al., 2006) showed no change in VO_2_max following 8 weeks of skill-based training, which was attributed probably to the low average HR (138 beats⋅min^–1^) achieved during volleyball training. However, the second study (Gabbett, 2008) found greater improvements for VO_2_max in adolescent boys and girls after skill-based conditioning games, which was due to higher intensity.

Some study limitations must be acknowledged. Overall physical activity of the students was not determined before and during the 8-month intervention period. However, they were advised to refrain from participating in any other physical activity during these 8 months. Furthermore, we did not control for dietary intake. However, most of the studies that had only assessed fitness components did not include dietary intake control. Moreover, another limitation is that we did not attempt to contextualize aggression (e.g., passive vs. reactive). However, the major strength of the study is that it was the first to investigate the effects on aggression parameters of school-based interventions comprising small-sided volleyball in 15-year-old adolescents. Boys showed better improvements in physical fitness compared to girls. Therefore, future studies should try to modify small-sided games in order to gain higher intensity, which may have a better impact on physical fitness in girls. Additionally, more tests related to health-related fitness should be used to provide a clearer picture of overall health and physical fitness level.

It can be concluded that the after school small-sided volleyball provides an appropriate stimulus for reducing aggression and improving physical fitness in high school students compared with standard PE classes. Girls showed greater reductions in aggressive behavior, while boys had better improvements in physical fitness. In contrast, the control group, which performed only PE classes, showed only small changes over the 8 months intervention. Our findings significantly contribute to the understanding of possible mechanisms for reducing adolescents’ aggression, which include enjoyment, motivation, and self-control.

## Data Availability Statement

All datasets generated for this study are included in the article/supplementary material.

## Ethics Statement

The studies involving human participants were reviewed and approved by the Institutional Ethics Committee from the Faculty of Sport and Physical Education, University of Novi Sad. Written informed consent to participate in this study was provided by the participants’ legal guardian/next of kin.

## Author Contributions

NT conceptualized the study design, recruited students into the study, and conducted the research. ŠB analyzed and interpreted the data and drafted the manuscript. MP conducted the research and drafted the manuscript. GS analyzed the data and conducted the research. LP interpreted the data and supervised the research. All authors contributed to the article and approved the submitted version.

## Conflict of Interest

The authors declare that the research was conducted in the absence of any commercial or financial relationships that could be construed as a potential conflict of interest.
